# A case of intravascular lymphoma diagnosed with a primary vitreoretinal lymphoma-like fundus lesion

**DOI:** 10.1186/s12348-021-00280-0

**Published:** 2021-12-24

**Authors:** Masaki Asakage, Kazuhiko Umazume, Hiroyuki Takoi, Daigo Akahane, Yasunori Ishibashi, Hiroshi Yamaguchi, Masahide Gondo, Hiroshi Goto

**Affiliations:** 1 Department of Ophthalmology, Tokyo Medical University, 6-7-1 Nishi-shinjuku, Shinjuku-ku, Tokyo, 160-00023 Japan; 2 Department of Respiratory Medicine, Tokyo Medical University, Tokyo, Japan; 3 Department of Hematology, Tokyo Medical University, Tokyo, Japan; 4 Department of Anatomic Pathology, Tokyo Medical University, Tokyo, Japan; 5 Department of Dermatology, Tokyo Medical University, Tokyo, Japan

**Keywords:** INTRAVASCULAR lymphoma, Vitreoretinal lymphoma

## Abstract

**Purpose:**

We report a case of intravascular lymphoma with primary vitreoretinal lymphoma-like fundus findings.

**Case:**

A 61-year-old man with a one-week history of temporal visual field defect in the left eye was referred by a local ophthalmologist to our department. A yellowish-white raised patchy lesion was found in the nasal fundus of the left eye. Vitreoretinal lymphoma was suspected, and vitrectomy was performed in the left eye for diagnostic purpose. However, vitreous interleukin-10 concentration was low and no significant result was obtained. He had fever of around 38 °C, and respiratory failure that started 2 weeks before ophthalmological examination, worsened. Intravascular lymphoma was diagnosed from the results of histopathological examinations of transbronchial lung biopsy, bone marrow biopsy and random skin biopsy. With the start of systemic chemotherapy, the subretinal lesions shrank gradually and systemic condition was stable. However, 5 months after the start of chemotherapy, spread to the central nervous system was observed, and chimeric antigen receptor T cell (CAR-T) therapy was started in another hospital. After the start of CAR-T therapy, the subretinal lesions shrank further.

**Conclusions:**

Intravascular lymphoma may be accompanied by primary vitreoretinal lymphoma-like intraocular lesions. If intraocular lesions are accompanied by systemic symptoms such as fever of unknown origin, the possibility of intravascular lymphoma should be suspected and systemic work-up should be performed.

## Introduction

Vitreoretinal lymphoma (VRL) is a rare malignancy arising in the vitreous and subretinal space, and may present before, after, or simultaneously with central nervous system spread. Diagnosis is based on ophthalmoscopic findings, interleukin (IL)-10/IL-6 ratio, immunoglobulin H receptor rearrangements and cytologic examination of vitreous biopsy [1].

Intravascular lymphoma (IVL) is characterized by proliferation of tumor cells in small blood vessels [2], and is a rare lymphoma that is difficult to diagnose due to diverse systemic symptoms such as fever of unknown origin and vascular occlusion [3].

We report a case of IVL diagnosed by investigation of VRL-like fundus findings.

## Case presentation

**Fig. 1:**
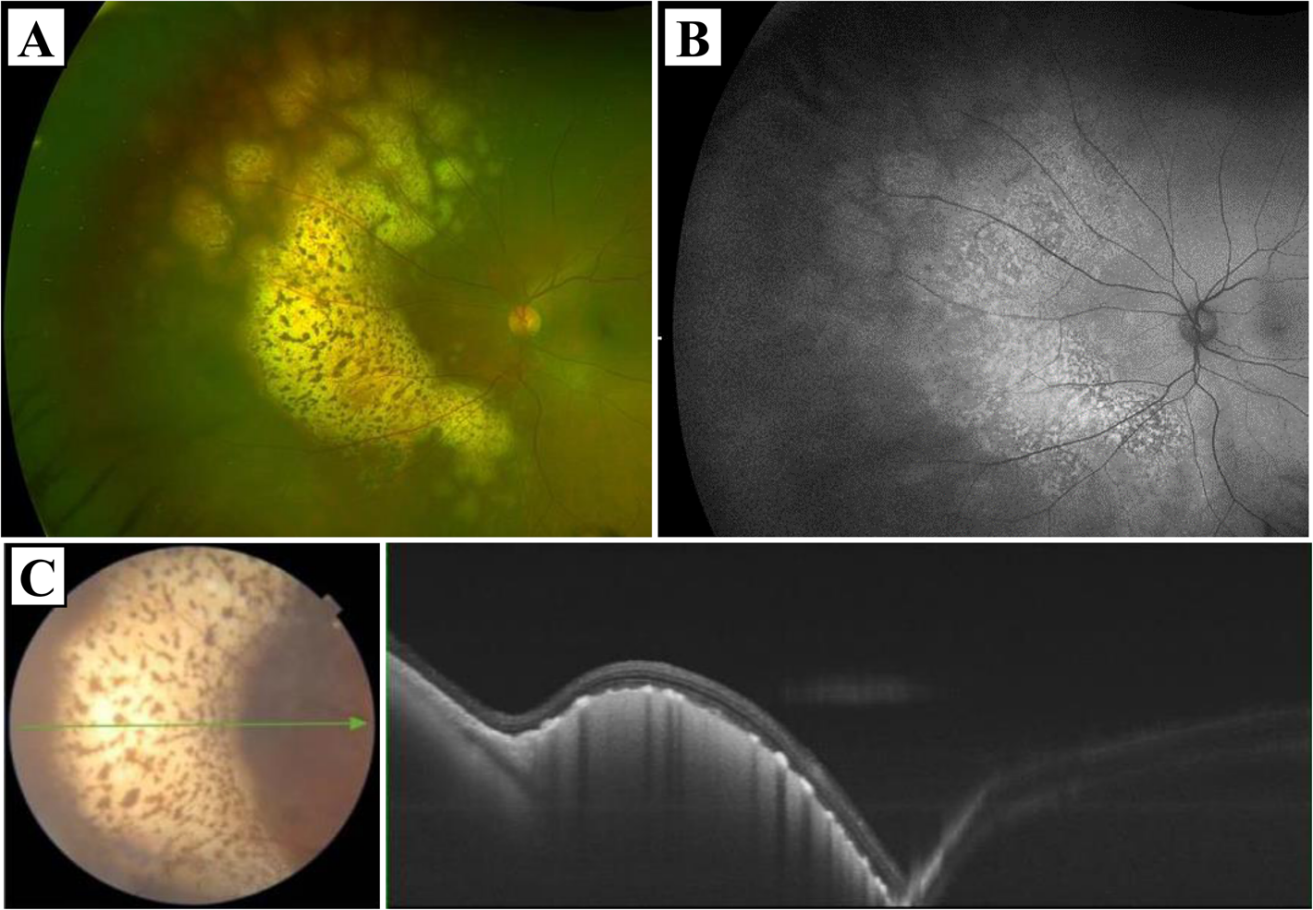
Imaging findings of the left eye at the first visit. (A) Fundus photograph showed a yellowish-white raised patchy lesion at the nasal fundus of the left eye. (B) In fundus autofluorescence imaging, a mixture of hyperfluorescence and hypofluorescence was observed at the same site. (C) Optical coherence tomography depicted lesions below the retinal pigment epithelium. The image was acquired along the green arrow

A 61-year-old man with a one-week history of temporal visual field defect in the left eye was referred by a local ophthalmologist to our department for investigation of white nasal retinal lesions. The patient had a low-grade fever of around 37 °C and mild respiratory failure for about 2 weeks before he was aware of visual field defects. At presentation, his best-corrected visual acuity was 24/20 in both eyes, and intraocular pressure was 10/11 mmHg. Slit-lamp examination revealed no specific findings other than mild cataracts. No inflammatory cells and vitreous opacity were observed. Fundus examination showed no abnormal findings in the right eye, but revealed a yellowish-white raised patchy lesion in the nasal fundus of the left eye. Fundus autofluorescence imaging of the same site showed a mixture of hyperfluorescence and hypofluorescence regions. Optical coherence tomography depicted a ridge in the retinal pigment epithelium and a subretinal lesion (Fig. 1). Laboratory findings were as follows: white blood cell count 6100/μL, hemoglobin 14.3 g/dL, platelet count 28.3 × 10^4^/μL, aspartate transaminase 84 U/L, alanine transaminase 21 U/L, blood urea nitrogen 12.9 mg/dL, creatinine 0.83 mg/dL, C-reactive protein (CRP) 0.60 mg/dL (reference range ≤ 0.30 mg/dL), lactate dehydrogenase (LDH) 923 U/L (reference range 106–211 U/L), soluble IL-2 receptor (sIL-2R) 609 U/L (reference range 122–496 U/L), and beta 2-microglobulin 2.02 mg/L (reference range 0.64–1.56 mg/L). VRL was suspected. Head magnetic resonance imaging (MRI) performed for the detection of central nervous system lesions revealed old cerebral infarction, but no lymphoma infiltration. On day 13 after the first visit, the patient was admitted to undergo phacoemulsification, intraocular lens implantation and 25-gauge pars plana vitrectomy for diagnostic purpose. Intraoperative examination revealed only mild vitreous opacity, and routine vitrectomy was performed. Immediately after vitrectomy, intravitreal injection of methotrexate (MTX) was started for suspected VRL. He was discharged the next day.

**Fig. 2:**
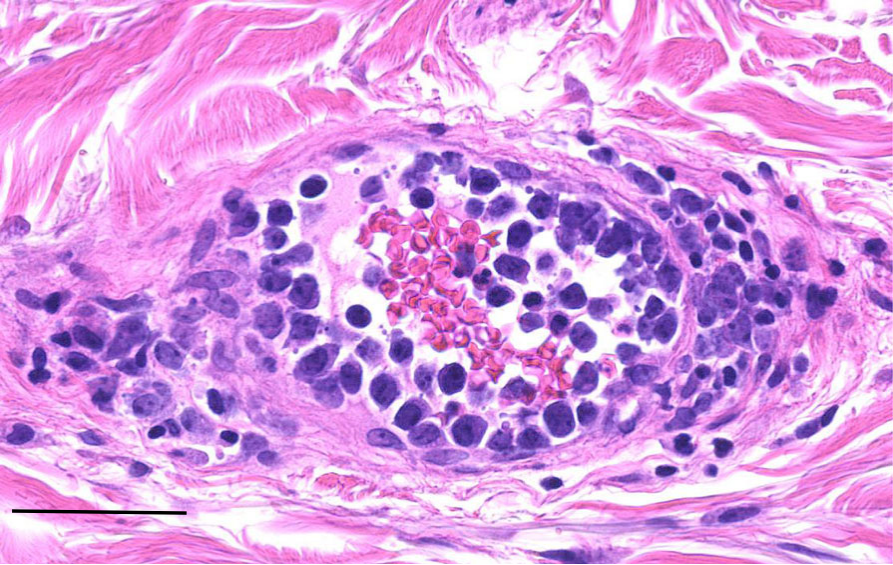
Histopathological findings of random skin biopsy. Many lymphocyte-like atypical cells were distributed in small blood vessels, and some were distributed in the dermis and subcutaneous adipose tissue (magnification 400×, bar: 50 μm)

The patient’s systemic symptoms did not resolve. One week after discharge, fever persisted at around 38 °C and respiratory failure worsened. Whole-body computed tomography (CT) was performed for detailed examination. Interstitial pneumonia and splenomegaly were found on CT, and he was admitted to the department of respiratory medicine of our hospital for detailed examination and treatment. LDH, CRP, sIL-2R, which were higher at the time of admission to respiratory medicine than at the first visit ophthalmology, further increased to 1381 U/L, 9.6 mg/dL, and 5239 U/mL, respectively. Changes in the above blood levels and histopathological examination of a transbronchial lung biopsy suggested IVL. For definitive diagnosis, bone marrow biopsy and random skin biopsies of seven sites (skin without apparent lesion in the left upper arm, skin with spider angioma-like lesion in the left anterior chest, left thigh, and left lower leg, and skin with cherry angioma lesion in the right shoulder, right anterior chest, and right lower leg) were additionally performed. Histopathological examination of the random skin biopsy showed many lymphocyte-like atypical cells distributed in small blood vessels, and some were distributed in the dermis and subcutaneous adipose tissue, confirming the diagnosis of IVL (Fig. 2).

**Fig. 3:**
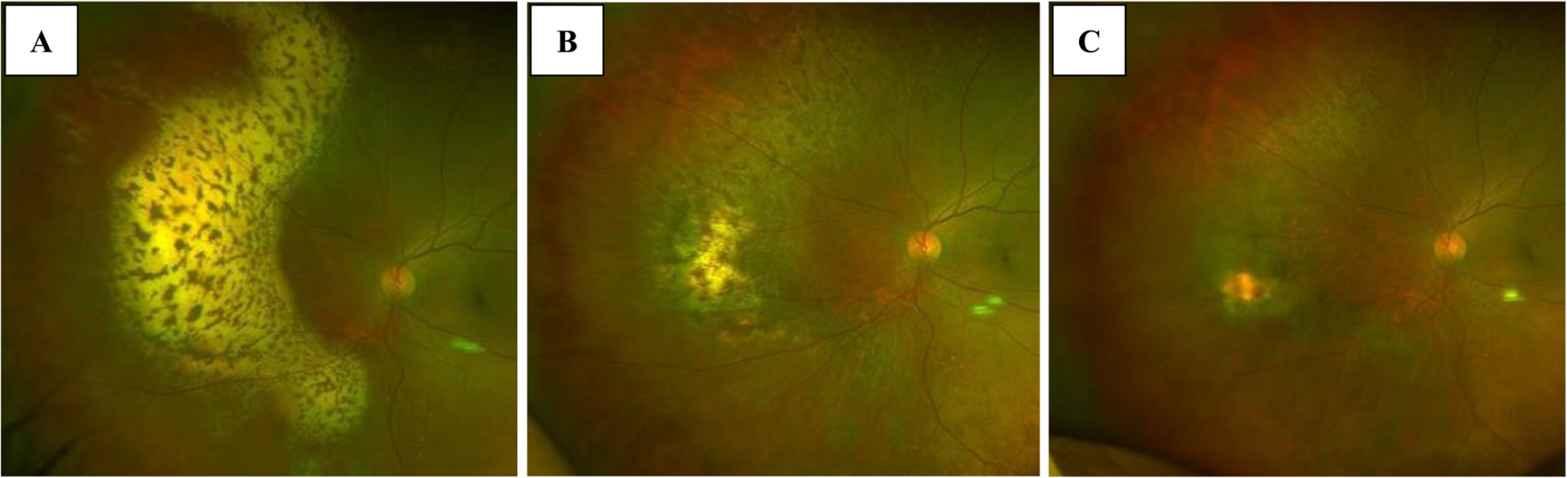
Fundus findings after topical and systemic treatment. (A) After 3 intravitreal MTX injections, the peripheral lesions tended to shrink. (B) Four months after the start of chemotherapy, the major lesion remained partially regressed. (C) After initiation of CAR-T therapy, the lesion shrank further

**Fig. 4:**
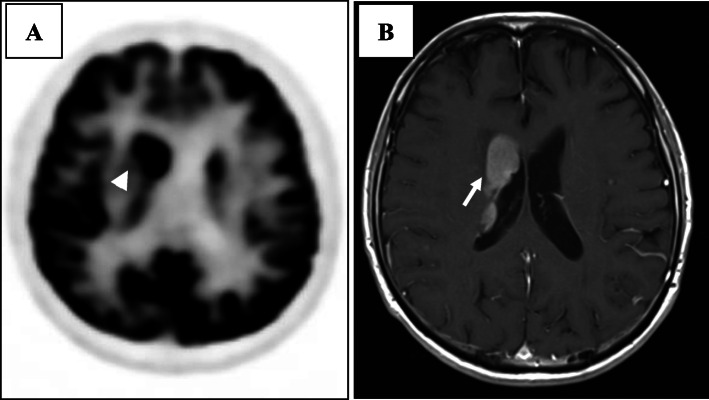
Head imaging at the time of central nervous system spread. (A) PET-CT showed abnormal accumulation in the right frontal lobe (arrow head). (B) Contrast-enhanced MRI (T1) showed an enhancing effect at the same site (arrow)

Regarding treatment, after vitrectomy was performed on day 13 after the first visit, intravitreal injection of MTX had been performed three times according to the treatment protocol for VRL, and the yellowish-white lesions in the peripheral retinal area tended to shrink (Fig. 3A). Since the fundus lesion was typical of VRL and vitreous IL-6 level was 198 pg/ml and IL-10 was 41 pg/m, which were poor findings for VRL, intravitreal MTX injection was scheduled to be continued. However, due to exacerbation of systemic symptoms, systemic treatment was started and intravitreal MTX injection was discontinued. Rituximab plus cyclophosphamide, doxorubicin, vincristine, and prednisone (R-CHOP) therapy and high-dose methotrexate (HD-MTX) therapy were started for the treatment of IVL at the department of hematology of our hospital. With the start of treatment, LDH, CRP and sIL-2R began to decrease and fever tended to improve. A total of six courses of R-CHOP therapy and two courses of HD-MTX therapy were performed within 4 months. Both systemic symptoms and laboratory findings tended to improve, and intraocular lesions also tended to shrink (Fig. 3B). Two more doses of rituximab were administered. However, positron emission tomography-CT performed 5 months after the start of chemotherapy showed abnormal accumulation in the right frontal lobe, and contrast-enhanced MRI showed an enhancing effect at the same site. Although the disease had spread to the central nervous system (Fig. 4), no central nervous system symptoms were observed. Then, chimeric antigen receptor T cells (CAR-T) therapy was started for the central nervous system lesion in another hospital. The retinal lesions did not worsen during central nervous system spread, and improved further after switching to CAR-T therapy (Fig. 3C). At the last follow-up, CAR-T therapy was successful, and systemic and intraocular findings remained favorable.

## Discussion

IVL manifests diverse symptoms such as fever of unknown origin, symptoms associated with vascular phagocytosis syndrome [4, 5], and symptoms associated with vascular occlusion. However, to the best of our knowledge, there are no reports of intraocular lesions, and this is the first report of IVL with VRL-like fundus lesion. IVL is a subtype of diffuse large B-cell lymphoma (DLBCL) and is defined as the proliferation of neoplastic lymphocytes in small blood vessels [2]. The retina and choroid are tissues rich in blood vessels, while the vitreous body is an avascular tissue. Besides, DLBCL is the most common histopathological subtype of lymphoma arising in the eye as VRL [6]. The main lesion in this case was subretinal, and it is possible that the lesion with VRL-like findings represents a phenotype of IVL expressed when located only under the retina without vitreous opacification.

Intravitreal MTX injection is generally an effective treatment for VRL [7]. In this case, systemic symptoms worsened during treatment with intravitreal MTX injection, resulting in discontinuation of this treatment. Since the treatment period was short, the therapeutic effect of MTX on the intraocular lesion of IVL in this case cannot be determined. In general, the combination of R-CHOP therapy and high-dose MTX therapy reduces the risk of developing the central nervous system [8]. As the retinal lesions were reduced slightly after intravitreal MTX injection, this treatment modality is considered to be also effective for intraocular lesion due to IVL. On the other hand, onset of central nervous system lesion was detected despite the above treatments. In general, VRL is associated with central nervous system involvement [1], which may reflect the characteristics of VRL. Compared to IVL without ocular lesion, greater attention may be required to detect development of central nervous system lesion in IVL with ocular symptoms.

IVL is difficult to diagnose because it manifests diverse symptoms depending on the affected organ, and there are many cases of postmortem diagnosis [9]. On the other hand, reports have shown that initiation of R-CHOP therapy increases the rate of complete remission and prevents recurrence [10] . Also, random skin biopsy used in the present case has been reported to be the most useful method for diagnosis [11, 12]. Random skin biopsy allows detection of pathological findings from sites without apparent skin symptoms. In the present case also, IVL lesions were detected both from sites with skin symptoms and sites without skin symptoms, indicating the usefulness of random skin biopsy. IVL is characterized by elevated LDH and sIL-2R [10]. In this case as well, these blood levels increased to a maximum of 1440 U/L and 8830 U/L, respectively.

The lack of previous reports may suggest that IVL manifesting ocular symptoms is rare. In the present case, in addition to systemic symptoms such as fever of unknown origin, blood tests revealed elevated LDH and sIL-2R, which may lead to a suspicion of IVL. In the case where vitreous biopsy findings are nonspecific, IVL can be diagnosed by actively performing skin biopsy. Furthermore, there is a possibility that life can be saved by close collaboration with the internal medicine department.

In this case, since CAR-T therapy was initiated in another hospital, tracking of the detailed clinical course of systemic findings is not possible. Therefore, there is limitation to observing the relationship between systemic findings and ocular findings.

## Data Availability

The datasets used and/or analyzed during the current study are available from the corresponding author on reasonable request.

## Abbreviations

VRL: Vitreoretinal lymphoma
IL: Interleukin
IVL: Intravascular lymphoma
CRP: C-reactive protein
LDH: Lactate dehydrogenase
sIL-2R: Soluble interleukin 2 receptor
MRI: Magnetic resonance imaging,
CT: Computed tomography
MTX: Methotrexate
RCHOP: Rituximab plus cyclophosphamide, doxorubicin, vincristine, and prednisone
HD-MTX: High-dose methotrexate
CAR-T: Chimeric antigen receptor T cells
DLBCL: Diffuse large B-cell lymphoma
